# 

**Published:** 1872-10

**Authors:** 


Vol. VIII. OCTOBER, 1872. No. 3.

### THE BISTOURY.

### A QUARTERLY JOURNAL,

*Devoted to the Exposition of Charlatanism in Medicine and the Education of the People upon Medical Subjects!*

Thad S. Up de Graff, M. D., Editor.

*Terms of Subscription, Fifty Cents per annum, in Advance.*

ELMIRA, N. Y.

PRINTED FOR THE PROPRIETOR, AT THE DAILY ADVERTISER JOB PRINTING HOUSE.

